# MicroRNAs Are Involved in Maize Immunity Against *Fusarium verticillioides* Ear Rot

**DOI:** 10.1016/j.gpb.2019.11.006

**Published:** 2020-06-10

**Authors:** Zijian Zhou, Yan Cao, Tao Li, Xinghao Wang, Jiafa Chen, Hang He, Wen Yao, Jianyu Wu, Huiyong Zhang

**Affiliations:** *1* State Key Laboratory of Wheat and Maize Crop Science, Collaborative Innovation Center of Henan Grain Crops, College of Agronomy, Henan Agricultural University, Zhengzhou 450002, China; *2* State Key Laboratory of Wheat and Maize Crop Science, Collaborative Innovation Center of Henan Grain Crops, College of Life Sciences, Henan Agricultural University, Zhengzhou 450002, China; *3* School of Advanced Agricultural Sciences and School of Life Sciences, State Key Laboratory of Protein and Plant Gene Research, Peking University, Beijing 100871, China

**Keywords:** Maize, MicroRNA, *Fusarium* ear rot, Deep sequencing, Disease resistance

## Abstract

***Fusarium* ear rot** (FER) caused by *Fusarium verticillioides* is one of the most common diseases affecting **maize** production worldwide. FER results in severe yield losses and grain contamination with health-threatening mycotoxins. Although most studies to date have focused on comprehensive analysis of gene regulation in maize during defense responses against *F. verticillioides* infection, less is known about the role of **microRNAs** (miRNAs) in this process. We used deep sequencing to compare small RNA libraries from the maize kernels of susceptible (N6) or resistant (BT-1) inbred lines from uninfected plants and upon *F. verticillioides* infection. We found that pathogen exposure was accompanied by dynamic alterations in expression levels of multiple miRNAs, including new members of previously annotated miRNA families. A combination of transcriptomic, degradomic, and bioinformatics analyses revealed that *F. verticillioides*-responsive miRNAs and their potential target genes displayed opposite expression patterns in the susceptible and resistant genotypes. Functional category analysis uncovered preferential enrichment of the pathogen-responsive miRNAs and their targets in the phenylpropanoid metabolic processes, plant–pathogen interactions, and plant phytohormone signal transduction pathways. Furthermore, transgenic maize plants overexpressing miR408b exhibited reduced resistance to *F. verticillioides* infection in a susceptible maize line. These findings provide new insights into the regulatory roles of miRNAs in maize immunity against FER and new resources for breeding **disease resistance** into maize.

## Introduction

*Fusarium* ear rot (FER) is a common fungal disease of the maize ear caused by *Fusarium verticillioides*. FER is responsible for considerable yield losses worldwide [1]. Symptoms of FER are related to plant genotype, environmental conditions, and the degree of infection [2]. Despite the erratic occurrence of FER epidemics, the disease needs to be controlled because the pathogen contaminates grain with harmful mycotoxins [3], [4]. The most effective strategies for overcoming FER are selecting resistant maize resources and developing pathogen-resistant varieties through breeding. Recently, next-generation precision genome engineering that targets defense mechanisms has been developed as an alternative to crop breeding techniques [5]. Therefore, it is crucial to explore the molecular mechanism underlying FER resistance in maize.

As sessile organisms, plants possess a sophisticated multi-layered immune system that protects them against microbial pathogens [6], including pathogen-associated molecular pattern (PAMP)-triggered immunity (PTI) and effector-triggered immunity (ETI). PTI and ETI rely on different signaling cascades, inducing a massive reprogramming of the plant transcriptome. The regulation of immune response genes in maize has been most extensively studied on the transcriptional level by next-generation sequencing and microarray approaches [7], [8], [9], [10]. However, to date, few efforts have been devoted to characterizing the potential role of microRNAs (miRNAs) in maize immunity against FER.

Accumulating evidence suggests that small RNAs play a vital role in plant immunity against pathogen attack [11], [12]. Small RNAs are classified as small interfering RNAs (siRNAs) or miRNAs, which mediate gene silencing either by directing DNA methylation or by directing mRNA degradation or translational repression [13]. Further, miRNAs are considered more diverse and active than siRNAs and attract more attention from researchers worldwide in the context of enhancing crop immunity against plant pathogens [14]. miR393 is the first small RNA identified to be involved in plant immunity, which actively contributes to PTI by repressing auxin signaling [15]. Previous studies have revealed the important roles of miR160a, miR398b, miR166, and miR773 in callose deposition, indicating their involvement in PTI immunity [16], [17], [18], [19]. Concerning ETI, distinct miRNA guiding cleavage of NBS-LRR-type disease resistance (R) genes has been described in Solanaceae and Leguminosae species, suggesting that miRNAs are key regulators in the ETI pathway [20], [21]. Furthermore, it has been shown that miRNAs function in crop immunity against fungal infection by targeting various genes. A number of miRNAs targeting disease resistance genes are considerably overexpressed in *Populus* infected by *Dothiorella gregaria* [22]. Yin et al [23] identified various miRNAs that endowed two cotton cultivars with resistance against *Verticillium dahliae*, and reduced levels of miR482 or miR1448 alongside corresponding increases in polyphenol oxidase (PPO) activities. In rice, a new miRNA, osa-miR7695, was shown to be involved in resistance to the blast fungus mycelia [24]. In addition, miR528 negatively regulated defense responses against rice stripe virus by directly decreasing expression of the gene encoding L-ascorbate oxidase (AO) [25]. Therefore, further investigations of miRNA-mediated regulatory processes in plant–pathogen interactions have considerable implications in developing new strategies for disease control and ultimately improving crop production.

In the current study, we aimed to identify miRNAs involved in maize immunity against FER. We performed a genome-wide screen by comparing the abundance of miRNAs in susceptible and resistant maize genotypes. High-throughput sequencing of six small RNA libraries from kernels that had been inoculated or not with *F. verticillioides* revealed a diverse collection of miRNAs, including known and new miRNAs, which were responsive to *F. verticillioides* infection. Functional characterization of the pathogen-responsive miRNAs and their targets revealed that they were preferentially involved in phenylpropanoid metabolic processes, plant–pathogen interactions, and plant phytohormone signal transduction pathways. Furthermore, overexpression of miR408b reduced the degree of *F. verticillioides* resistance in susceptible maize. These findings demonstrate that miRNA levels change with *F. verticillioides* infection and that it may be feasible to engineer FER resistance in maize by manipulating the expression of individual miRNAs.

## Results

### Two maize inbred lines, N6 and BT-1, display distinctly different FER resistance

**Figure 1:**
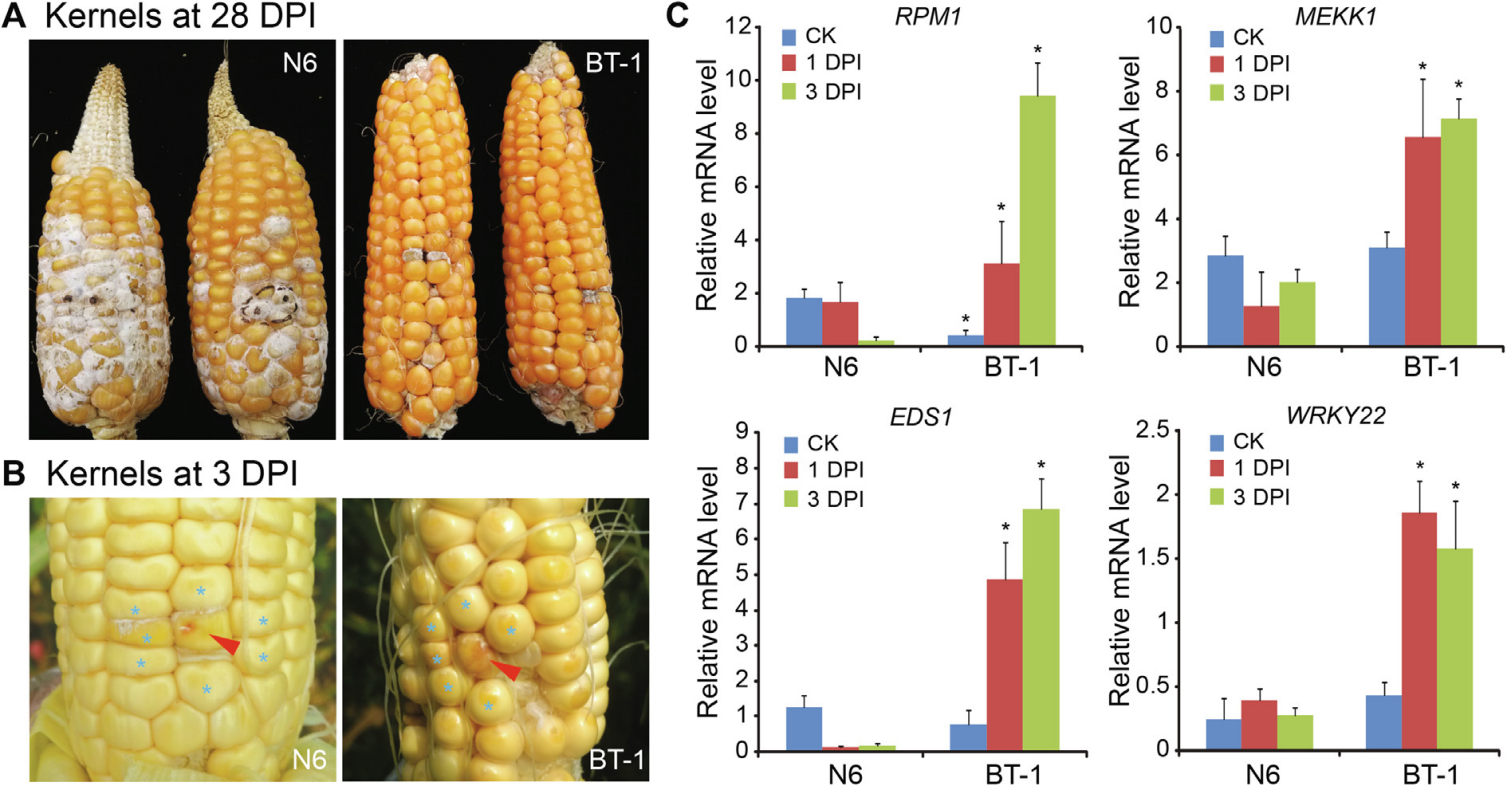
**Comparison of the defense responses to *F. verticillioides* in the susceptible (N6) and resistant (BT-1) maize inbred lines** **A.** Maize ears at 28 DPI. **B.** The maize kernels at 3 DPI. Maize kernels were inoculated by *F. verticillioides* at 15 days after pollination. For following analysis, kernels (as indicated with blue stars) close to the inoculated spots (red arrow) were collected at 1 DPI and 3 DPI for RNA extraction. **C.** Expression of the indicated defense-related genes in N6 and BT-1 upon *F. verticillioides* infection. Total RNA was extracted from the kernels as indicated in panel B for RT-qPCR analysis. Transcript levels were normalized to those in an untreated N6 line (control). Data are from three biological experiments. Asterisks indicate significant differences (*P* < 0.01, Student’s *t*-test). CK, control; DPI, day post inoculation.

To determine miRNAs that are most likely involved in maize immunity against FER, we analyzed two inbred lines identified as showing distinctly different FER resistance. The resistant line, BT-1, which originates from the Suwan germplasm of Thailand and the elite inbred line 8085 of China, displays strong defense responses to *F. verticillioides* infection. The susceptible line, N6, which is derived from HuangZaoSi, exhibits superior traits, such as good plant architecture, high combining ability, and stress resistance, but is highly susceptible to *F. verticillioides*. The resistant line displayed obvious resistance phenotypes either at the late or early stages of kernel development, compared with the susceptible line, upon *F. verticillioides* infection (Figure 1A and B). To further evaluate the defense responses of these two inbred lines, the maize kernels were inoculated with *F. verticillioides* 15 days after pollination*,* and kernels surrounding the inoculated points, as shown in Figure 1B, were collected 1 and 3 days post inoculation (DPI). We then examined the expression of four defense-related genes encoding the disease resistance protein (*RPM1*), mitogen-activated protein kinase kinase kinase 1 (*MEKK1*), enhanced disease susceptibility 1 (*EDS1*), and WRKY transcription factor 22 (*WRKY22*) by RT-qPCR. The transcript levels of all four genes were significantly increased in BT-1 plants compared with those in N6 plants upon infection (Figure 1C). Furthermore, expression of all four genes was induced in BT-1 plants but repressed in N6 plants (except for *WRKY22*) after exposure to the pathogen. Together, these observations suggest that the immunity against fungal invasion is greatly stimulated in BT-1 plants, resulting in a stronger defense response than in the N6 line. Therefore, we used these two maize inbred lines as models to identify miRNAs involved in defense immunity against FER.

### Deep sequencing of small RNA populations from *F. verticillioides*-free and infected maize kernels reveals a set of fungus-responsive miRNAs

Six small RNA libraries in total were prepared with maize kernels from the susceptible or resistant inbred lines treated (for 1 or 3 d) or untreated with *F. verticillioides* 15 days after pollination, and analyzed by high-throughput sequencing. After removing the sequencing adapters, low-quality reads, contaminants, and reads shorter than 18 nt or longer than 30 nt, about 13–15 million clean reads were successfully generated from six libraries, each representing different time points of both genotypes (Table S1, Sheet 1). By mapping to the maize genome (ZmB73_RefGen_v4), about 9–11 million maize small RNA reads were obtained. An Rfam search (http://xfam.org/) was performed with these mapped reads to filter the known small RNAs, such as rRNAs, tRNAs, and small nucleolar RNAs (snoRNAs). Consequently, 3–5 million reads were retrieved and regarded as small RNAs originating from maize. Further, 28% –35% of reads from each library were not matched to the maize genome (Table S1, Sheet 1), presumably due to DNA sequence polymorphisms between the maize cultivars and the reference genome, or *F. verticillioides* contamination in the infected maize kernels. However, very few reads in the six libraries were matched to the *F. verticillioides* 7600 genome (Table S1, Sheet 1), suggesting that the identified small RNAs indeed originated from maize.

The most abundant small RNAs are the 24-nt class in the unique reads (Figure S1A), while the known miRNAs are 21-nt long (Figure S1B). The clean reads were then aligned to all mature sequences of plant miRNAs deposited in the miRBase database (http://www.mirbase.org) to search for matches to the known miRNAs. This resulted in the identification of 258 known miRNAs belonging to 28 families (Figure S1D). Expression levels of miRNAs detected by Northern blotting were consistent with their frequencies in the sequencing dataset (Figure S2A). However, caution should be taken for the miRNAs with a low number of reads because they normally are not detected by Northern blot analysis.

The remaining unique reads were then used to identify novel miRNAs. Subsequently, 123 novel miRNAs (pre-miR001 to pre-miR123) were identified in the six libraries (Table S1, Sheet 2). The novel miRNAs were predominantly 21-nt and 24-nt long (Figure S1C), and started with a 5′-A (Table S1, Sheet 2). Although we have provided bioinformatics evidence for the 123 miRNA precursor structures, they have still been considered as miRNA candidates. Stem-loop RT-PCR was then performed to validate the novel miRNAs, and 9 of 12 predicted miRNAs were detected (Figure S2B), five of which were further validated by RNA blotting (Figure S2C). In addition, a high degree of complementarity for the precursor structures was observed (Figure S2D) and was consistent with a characteristic feature of young, recently evolved *MIR* genes [26].

**Figure 2:**
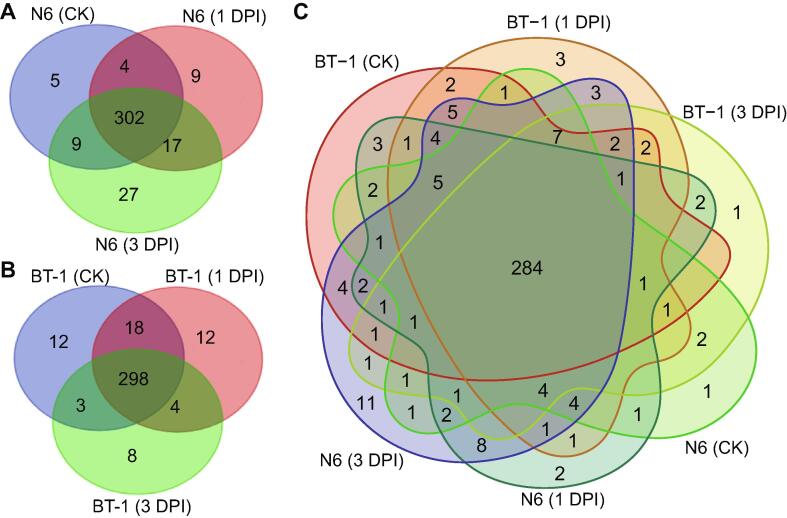
**Venn diagram showing miRNAs detected by deepsequencing of maize kernels exposed or not to *F. verticillioides*** **A.** Overall, 373 miRNAs were identified as expressed in the susceptible inbred line N6. **B.** Overall, 355 miRNAs were identified as expressed in the resistant inbred line BT-1. **C.** Venn diagram showing the distribution of the expressed miRNAs in all (six) samples, with 284 miRNAs considered common to both susceptible and resistant genotypes.

Next, we evaluated the miRNA distributions in the libraries of susceptible and resistant genotypes. In the susceptible genotype, five miRNAs (miR171m-5p, miR171a-5p, miR169l-5p, miR166i-5p, and pre-miR090) were specifically expressed in the control sample, while 53 miRNAs (*e.g.*, miR529-3p, miR2275c-5p, and miR2118g) were specifically expressed after pathogen treatment (Figure 2A; Table S1, Sheet 2). In addition, 302 miRNAs were commonly expressed in the control and infected samples (Figure 2A). For the resistant genotype, three libraries shared 298 miRNAs in common (Figure 2B). Twelve miRNAs (*e.g.*, miR172b-5p, miR393b-3p, and miR160g-3p) were specifically expressed in the control sample, whereas 24 miRNAs (*e.g.*, miR2275d-5p, miR396h, and miR2118c) showed specific expression in response to *F. verticillioides*. When comparing the six small RNA libraries containing 381 miRNAs, we identified 284 miRNAs that were expressed in all samples, while 32 miRNAs (26 in N6 plants and 8 in BT-1 plants) were expressed in a genotypic-dependent manner (Figure 2C).

### Some miRNAs identified in maize kernels specifically respond to *F. verticillioides*

Subsequent analysis of the high-throughput sequencing data indicated that *F. verticillioides* treatment was accompanied by alterations in the accumulation of a number of maize miRNAs. Specifically, 295 of 381 (77.4%) miRNAs from the six kernel libraries were differentially expressed, with fold change (FC) ≥ 1.5 and false discovery rate (FDR) ≤ 0.05 (Table S1, Sheet 3). Based on their change trends, these differentially expressed miRNAs (DEMs) were grouped into 40 patterns by using Short Time-series Expression Miner (STEM) [27]. Of these, miRNAs in 10 patterns displayed significant accumulation changes upon *F. verticillioides* infection (Table S1, Sheet 4; Figure S3). We further grouped the miRNAs in the 10 patterns into three classes, based on whether the accumulation change: (1) increased or decreased in both N6 and BT-1 plants upon *F. verticillioides* infection (patterns 8 and 39); (2) increased in N6 but decreased in BT plants (patterns 16, 18, 24, 28, and 31), or did not change obviously in BT-1 plants (pattern 25); and (3) decreased in N6 plants but did not change obviously in BT-1 plants (pattern 32), or decreased in N6 plants but increased in BT-1 plants (pattern 33). Theoretically, the first class of miRNAs may regulate the basal responses to *F. verticillioides* infection because of their up- or down-regulation in both susceptible and resistant maize lines, while the latter two classes of miRNAs would play negative and positive roles, respectively.

**Figure 3:**
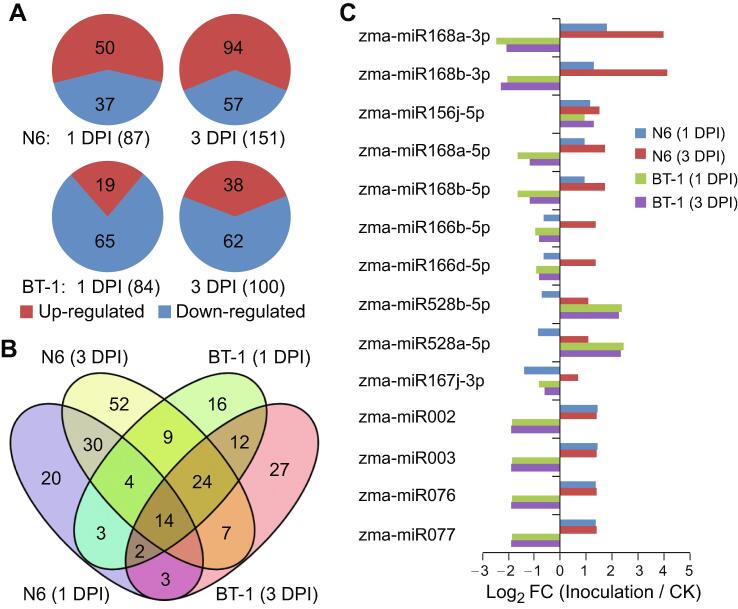
***F. verticillioides*-responsive miRNAs in the susceptible and resistant genotypes** **A.** Significantly expressed miRNAs in N6 (upper) and BT-1 (lower) lines infected with *F. verticillioides* for 1 or 3 D compared to CK samples. **B.** Comparison of *F. verticillioides*-responsive miRNAs in the susceptible and resistant genotypes upon infection. **C.** Expression profiles of *F. verticillioides*-responsive miRNAs common to the two genotypes, as determined by deep sequencing.

When comparing the genotypes separately, 87 and 151 DEMs were identified in 1 DPI and 3 DPI samples in the susceptible line, respectively (Figure 3A), the majority of which showed up-regulated expression upon *F. verticillioides* infection. In the resistant line, 84 and 100 miRNAs were differentially expressed at 1 DPI and 3 DPI, respectively (Figure 3A), expression of which were mostly down-regulated upon *F. verticillioides* infection. These observations indicate that miRNAs might regulate maize immunity against *F. verticillioides* invasion either at the early (1 DPI) or late (3 DPI) stages. Together, 234 miRNAs were designated as *F. verticillioides*-responsive in either the susceptible or resistant genotypes (Figure 3B; Table S1, Sheet 5), and displayed distinct expression patterns in response to infection (Figure 3C). In some cases, expression of a miRNA was generally consistent between two maize genotypes (*e.g.*, miR164a–g and miR156j), whereas in other cases, genotype-dependent response to *F. verticillioides* infection was observed (*e.g.*, miR168a,b, miR166b,c,d,h, and miR398a,b). Furthermore, there is a differential response at different time points of pathogen treatment for particular miRNAs (*e.g*., miR390ab and miR399ach; Table S1, Sheet 2).

We next analyzed the DEMs between the susceptible and resistant genotypes with or without pathogen infection. As shown in Figure S4A, 202, 186, and 186 miRNAs were differentially expressed in the control, 1 DPI and, 3 DPI samples, respectively, between these two lines. Compared to the susceptible line, expression of the majority of DEMs was up-regulated in the resistant genotype for the control samples, but down-regulated either quickly or long after the inoculation. In addition, 97 DEMs were commonly expressed in all the tested samples, and 29 miRNAs were genotype-specific (Figure S4B). Further, expression of 78 (24, 24, and 30) DEMs in N6 and BT-1 genotypes was specifically induced early (1 DPI) or late (3 DPI) during the infection, and the expression of 173 (41, 97, and 35) miRNAs was affected by both the genotype and infection. Collectively, 251 miRNAs were differentially expressed in the susceptible and resistant genotypes upon infection. Several DEMs were randomly selected for confirmation using RT-qPCR analysis, which shows good agreement between the sequencing data and RT-qPCR analysis (Figure S4C).

### Characterization of FER-related miRNAs in the susceptible and resistant maize lines suggests their role in FER resistance

**Figure 4:**
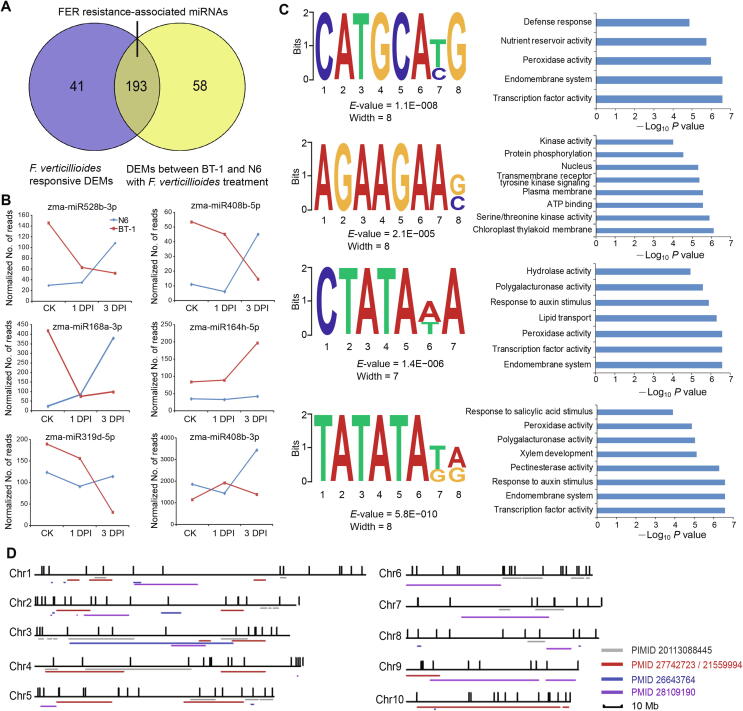
**Characteristics of FER resistance-associated miRNAs** **A.** 193 of miRNAs were identified to be associated with FER resistance between the susceptible and resistant maize lines. **B**. Expression profiles of representative FER resistance-associated miRNAs in the susceptible and resistant maize lines. **C.** Sequence motifs in the promoter regions of FER resistance-associated miRNAs (left) and GO terms of their associated transcription factors (right). The *y*-axis of the sequence logo represents information contents in bits. E values for the motifs were calculated by comparison with shuffled sequences. **D.** Distribution of FER resistance-associated miRNAs in the FER metaQTL in maize. The short horizontal lines represent the locations of anti-ear rot QTLs determined in previous studies (PMID provided). The short vertical lines represent the different types of FER resistance-associated miRNAs by STEM analysis. FER, *F. verticillioides* ear rot; DEM, differentially expressed miRNA; QTL, quantitative trait locus.

Although genetic variation might have resulted in specific miRNA differences between the susceptible and resistant genotypes, we could not exclude the possibility that these genotype-specific miRNAs were relevant to FER resistance because of their responsiveness to infection (Figure S4). Therefore, 193 miRNAs were tentatively assigned as FER resistance-related after considering the differential expression both in response to pathogen and between the two genotypes upon infection. These include 118 known and 75 predicted miRNAs (Figure 4A; Table S1, Sheet 6), which displayed distinct expression patterns in response to *F. verticillioides*. For example, some miRNAs (*e.g.*, miR528b-3p, miR408b-5p, and miR168a-3p) were expressed at significantly higher levels in uninfected kernels of the resistant genotype than in the susceptible genotype, but the levels decreased at 3 DPI (Figure 4B). In addition, the expression of several miRNAs (*e.g.*, miR164b-5p and miR319d-5p) did not change obviously regardless of *F. verticillioides* infection in the susceptible genotype but significantly increased or decreased in the resistant genotype. These observations suggest the possible regulation of maize immunity against *F. verticillioides* by miRNAs.

To explore whether the identified miRNAs share common and characteristic sequence elements, we performed an unbiased search for consensus motifs enriched in 2-kb regulatory regions immediately upstream of the stem-loop precursors. Thirty-five sequence motifs with biological functions were identified in the DEMs responsive to pathogen infection (Table S1, Sheet 7). Intriguingly, four of the motifs are specifically associated with defense response, transcription factor activity, peroxidase activity, and response to a plant hormone stimulus (Figure 4C). Furthermore, 118 of 193 DEMs were preferentially enriched in known quantitative trait loci (QTLs) associated with FER resistance in the maize genome (Figure 4D; Table S1, Sheet 8). Similarly, *F. verticillioides*-responsive miRNAs (138 of 234) as well as the DEMs (163 of 251) between the susceptible and resistant genotypes upon inoculation were also preferentially associated with the FER resistance QTLs (Figure S5; Table S1, Sheets 9 and 10). The potential association of *F. verticillioides*-responsive miRNA and FER-related QTLs further suggests that miRNAs might greatly contribute to FER resistance in maize.

### *F. verticillioides*-responsive miRNAs participate in plant–pathogen interaction and hormone signaling pathways

**Figure 5:**
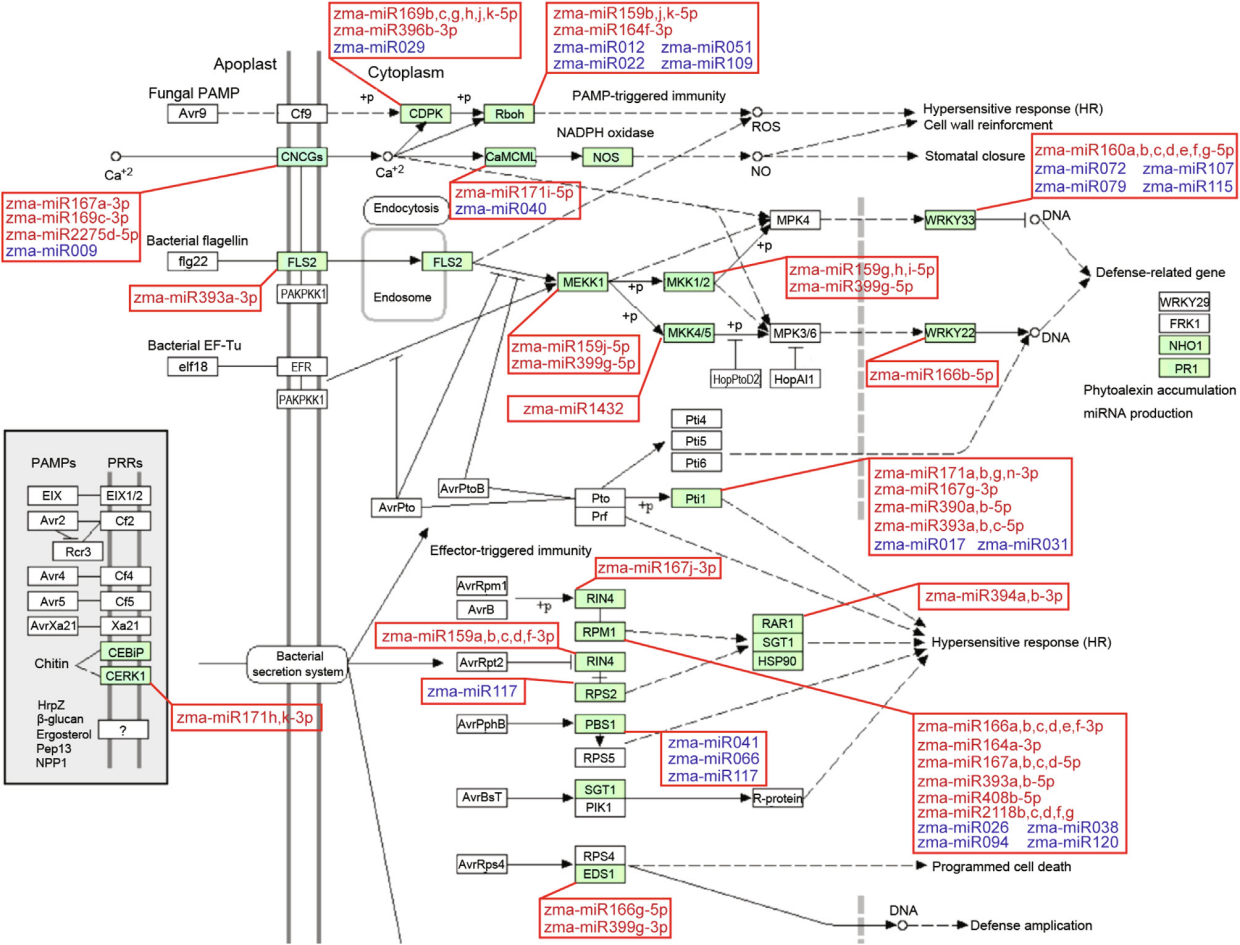
**Plant–pathogen interaction signal transduction pathway and the related miRNAs** Plant–pathogen interaction signal transduction pathway components are regulated by various miRNAs identified in the current study. The plant-pathogen interaction pathway was downloaded from the website (https://www.genome.jp/kegg-bin/show_pathway?zma04626), in which genes encoding the key components (green-colored) were explored to be potentially targeted by the corresponding miRNAs identified in the present study. Known miRNAs are denoted in red and predicted new miRNAs are indicated in blue.

The plant–pathogen interaction pathway (KEGG pathway zma04626; http://www.genome.jp) is represented by 208 genes and comprises two defense mechanisms (PTI and ETI) against pathogen invasion. Interestingly, a number of pathogen-responsive miRNAs, including 68 known and 21 predicted miRNAs, were involved in this pathway (Figure 5). For instance, MAPK signal transduction plays an important role in the primary response of the PTI pathway, genes encoding many components of which, such as calcium-dependent protein kinase (*CDPK*), respiratory burst oxidase protein B (*RBOH*), LRR receptor-like serine/threonine-protein kinase (*FLS2*), and WRKY transcription factor 33 (*WRKY33*), were targeted by various miRNAs (*e.g.*, miR396b-3p, miR164f-3p, miR171i-5p, miR2275d-5p, miR399g-5p, and miR160a-5p; Figure 5). Similarly, genes encoding disease resistance analog PIC11 isoform X1 (*RPM1*), serine/threonine-protein kinase (*PBS1*), and pto-interacting protein 1 (*PTI1*) from the ETI pathway were regulated by miR408b-5p, miR164a-3p, miR159a-3p, miR166b-3p, and miR390a-5p (Figure 5).

To further validate whether the aforementioned *F. verticillioides*-responsive miRNAs are involved in the plant–pathogen interaction, we selected three known miRNAs (miR166b, miR396b, and miR408b) for analysis. We compared their expression levels as well as those of their predicted targets in six representative maize inbred lines, namely, three FER susceptible (N6, TSTP21, and TSTP12) and three resistant (BT, CML533, and CML325) lines. As shown in Figure S6, the miRNAs and their potential targets displayed the similar expression trends in various susceptible or resistant genotypes. For instance, the expression of miR166b, upregulated in the N6 line and downregulated in the BT-1 line upon *F. verticillioides* treatment, was significantly (*P* < 0.05) increased in the other two susceptible lines (TSPT21 and TSTP12) and repressed in the resistant lines (CML533 and CML325), respectively.

Plant defense responses are induced via the phytohormone pathway [28]. It was therefore not surprising that FER resistance-related miRNAs regulate these signaling processes (Figure S7). For instance, phenylalanine biosynthesis contributes to salicylic acid (SA) signaling, and several miRNAs (*e.g.*, miR2275b/c, miR164b, and miR166g) were found to target genes encoding the key components of this process, *e.g.*, regulatory protein NPR1, transcription factor TGA, and pathogenesis-related protein 1 (PR1). In addition, genes encoding ethylene receptor (*ETR*), ethylene-insensitive protein 2 (*EIN2*), and ethylene-responsive transcription factor 1/2 (*ERF1/2*) of the ethylene signaling pathway, were targeted by miR166a-3p, miR159h-3p, miR169l-3p, and miR171g-5p. These observations demonstrate that the interaction of pathogen-responsive miRNAs and multiple signaling pathways plays an essential role in immunity against FER in maize.

### Degradome and target prediction analyses reveal specific roles of enriched miRNAs and their targets

Integrative analysis of miRNAs and their target genes can provide insight into the regulatory pathways of miRNAs, and determine functional miRNA–mRNA modules related to FER resistance. We applied degradome sequencing to identify miRNA targets in maize kernels. Overall, 324 specific miRNA–mRNA interactions were predicted at cleavage sites (*P* < 0.05; Table S2), with 80 cleavage sites determined, as well as 61 target genes associated with 118 miRNAs, including two novel miRNAs (pre-miR079 and pre-miR115). Among the target genes, 15 of them responsive to stimuli, such as auxin response factor (ARF) and ARF F-box proteins, were the most significant and cleaved by miR160 and miR167 families, as well as by two novel miRNAs (Table S2). Transcription factors represented another important class of cleavage peaks, with genes such as *MYB*, *NAC*, *SPL*, *TCP*, and *ATHB4*, cleaved by miR159, miR164, miR156, miR319, and miR166 families, respectively. Interestingly, the expression of miR168a-5p or miR168b-5p that target the key component of the gene silencing pathway, *AGO1*, was significantly (*P* < 0.05) increased in the susceptible genotype but decreased in the resistant genotype after infection (Table S1, Sheet 2). Consistent with this observation, expression levels of *AGO1* were dramatically decreased in the RNA sequencing datasets of the susceptible genotype and increased in the resistant genotype upon *F. verticillioides* infection (Table S4). Thus, it is speculated that a fine-tuned adjustment of miR168 levels by host plants would help to maintain appropriate levels of AGO1 to ensure a proper balance of other miRNAs during the maize response to *F. verticillioides* invasion.

Considering the limited number of miRNA targets identified by degradome analysis, psRNATarget [29] was next used to predict the potential target genes for a comprehensive understanding of miRNA function during infection. Consequently, 22,880 miRNA–target pairs were identified, representing 2877 distinct maize genes (Table S3). Gene Ontology (GO)-based enrichment analysis indicated that these targets were associated with various biological functions (Table S1, Sheet 11), such as the phenylpropanoid metabolic process, lignin catabolic process, regulation of phosphorylation, and response to hormone stimulus (Figure S8A). Furthermore, KEGG pathway analysis revealed that 38 KEGG pathways were significantly (*P* < 0.05) enriched for the target genes. The biosynthesis of secondary metabolites, ABC transporters, peroxisome, oxidative phosphorylation, transcription factors, and plant–pathogen interaction were among the most significantly enriched annotated pathways (Figure S8B; Table S1, Sheet 12).

### *F. verticillioides*-responsive miRNAs showed a negative correlation with the expression of their respective targets

Global transcript profiling was then performed to investigate whether the changes in the expression of target genes were accompanied by inverse changes in the expression of *F. verticillioides*-responsive miRNAs. We conducted RNA sequencing of the control and 3 DPI kernels from the susceptible and resistant genotypes (Table S4). A series of genes were identified to be significantly affected (up- and down-regulated) in response to *F. verticillioides* inoculation (FDR < 0.05; FC ≥ 1.2). Further analysis revealed a substantial number of target genes showing opposite expression changes to that of their corresponding regulatory miRNAs, either in the susceptible or resistant genotype, upon infection (Figure S9), suggesting a functional interaction in the regulation of FER resistance. However, such opposite expression patterns were not apparent for some miRNA–target pairs, perhaps indicating that the target genes might be translationally repressed or display a dynamic response to the pathogen.

**Figure 6:**
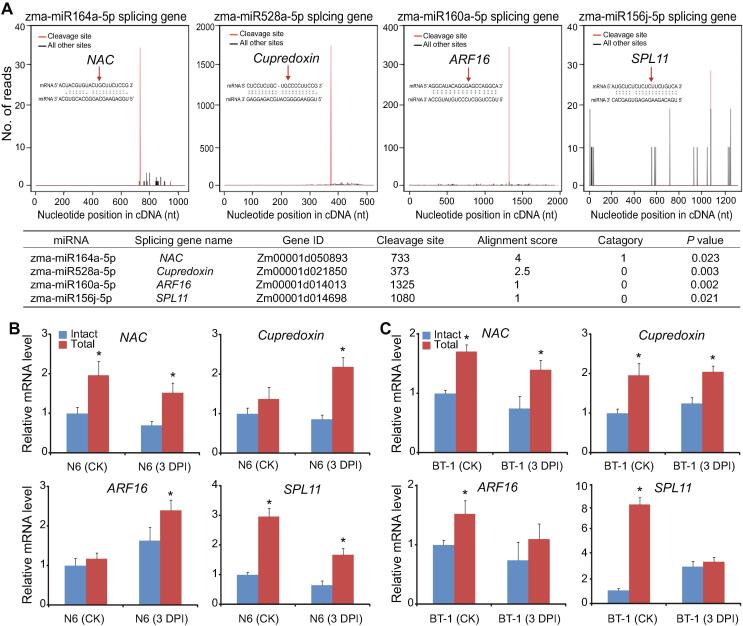
**Analysis of miRNA target genes** **A.** Target plots and miRNA–mRNA alignments in maize determined by degradome sequencing. Genes *NAC*, *Cupredoxin*, *ARF16*, and *SPL11* are cleaved by miR164a-5p, miR528a-5p, miR160a-5p, and miR156j-5p, respectively. The red arrows represent the cleavage nucleotide positions in the target genes. **B.** RT-qPCR analysis of intact or uncleaved and total mRNA levels in maize kernels from the susceptible N6 line. **C.** RT-qPCR analysis of intact and total mRNA levels in maize kernels from the resistant BT-1 line. Values represent means ± SD of three biological replicates. *, *P* < 0.05 (two-tailed Student’s *t*-test).

To further validate the cleavage of mRNAs by miRNA during pathogen invasion, we used RT-qPCR with two pairs of primers designed for the 3′-UTR of target mRNA and spanning the miRNA-binding site to detect the total mRNA and intact or uncleaved mRNA, respectively, of four target genes identified by the degradome analysis (Figure 6A). As shown in Figure 6B, the total mRNA of the four genes showed similar change trends with that of the intact mRNA except for *Cupredoxin* targeted by miR528a-5p in the susceptible line. Meanwhile, increased cleavage of the *NAC*, *Cupredoxin*, *ARF16*, and *SPL11* transcripts (targeted by miR164a-5p, miR528a-5p, miR160a-5p, and miR156i-5p, respectively) was observed, in agreement with a significant increase of the four miRNAs after pathogen inoculation in the susceptible line. By contrast, decreased cleavage of *ARF16* and *SPL11* mRNAs was observed in the resistant line upon pathogen treatment, which was consistent with the lack of obvious changes in the corresponding miRNA levels (Figure 6C).

### Zma-miR408b directly participates in *F. verticillioides* resistance

**Figure 7:**
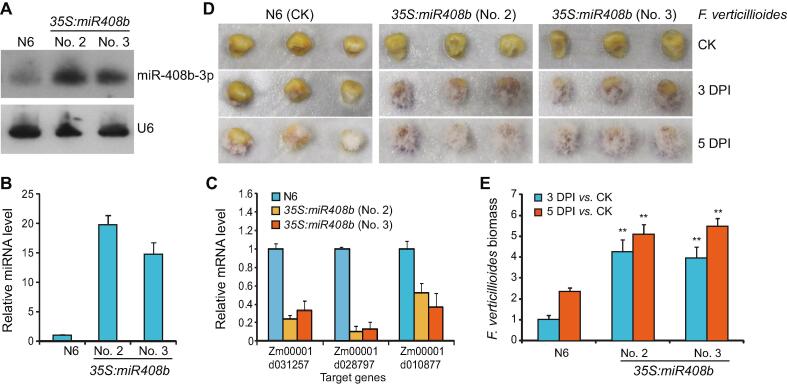
**miR408b affects the degree of resistance to *F. verticillioides* of the susceptible maize genotype RNA blot analysis** **A** and RT-qPCR analyses **B** performed to examine the accumulation of miR408b in the kernels of the indicated transgenic or control plants. **C.** RT-qPCR analysis of the three indicated target genes of miR408b in the indicated transgenic lines. RNA was extracted from the T2 generation of *35S:miR408b* transgenic plants. **D.** Sensitivity to *F. verticillioides* infection of kernels from the transgenic plants 5 weeks after pollination. The kernels were inoculated with a spore suspension of *F. verticillioides* (5 × 10^5^ spores/ml), and the plants were photographed 3 and 5 DPI. **E.** RT-qPCR analyses of *F. verticillioides* biomass (based on the expression level of FVEG_04081) on the indicated maize genotypes at 3 or 5 DPI. Maize *Ubiquitin 2* gene was used as an internal control. Values represent means ± SD of three independent samples. *, *P* < 0.01 (two-tailed Student’s *t*-test).

To gain further insight into the potential function of the identified FER resistance-associated miRNAs, transgenic maize plants overexpressing the precursor, *MIR408b*, were next tested. miR408b is a negative regulator of resistance, and its levels changed appreciably in the resistant genotype but increased approximately 2-fold after infection in the susceptible genotype (Table S1, Sheet 2). Two independent transgenic maize lines with high miR408b levels were obtained and characterized by both RT-qPCR and RNA gel blot analyses (Figure 7A and B). Consistently, the expression of the target genes Zm00001d031257 (encoding cupredoxin), Zm00001d028797 (encoding laccase 13), and Zm00001d010887 (encoding serine/threonine-protein phosphatase BSL1), was significantly reduced compared with the control line (Figure 7C). Next, after 5 weeks of pollination, we inoculated the transgenic maize kernels with an *F. verticillioides* spore suspension, and fungal growth phenotypes were recorded after 3 and 5 days. As shown in Figure 7D, the disease symptoms in transgenic plants overexpressing *MIR408b* were obviously more pronounced than those in the wild-type maize (N6 line). The obvious growth of fungal hyphae was observed with both transgenic lines but was not as apparent in the control samples on 3 DPI, and fungal hyphae covered the surface of the tested transgenic kernels on 5 DPI (Figure 7D and E). These observations indicate that the expression of miR408b affects the susceptibility of the genotype susceptible to *F. verticillioides* infection.

## Discussion

Host (plant) miRNAs play vital roles in defense responses against pathogens by modulating transcriptome reprogramming at the post-transcriptional level. Characterization of host miRNAs that are responsive to infection is the first step of exploring their function in host immunity against a pathogen. Increasing number of miRNAs with essential regulatory roles in plant immunity against fungal pathogens have been discovered in various plant species [11], [18], [19], [22], [23], [30], [31], [32]. However, few efforts have been devoted to characterizing the roles of miRNAs in maize against fungal pathogens. In the current study, using deep sequencing, we showed that expression levels of a number of miRNAs exhibited dynamic alterations in maize kernels from two distinct inbred lines in response to *F. verticillioides* treatment.

The majority of these known miRNAs appears to negatively regulate immunity of maize against *F. verticillioides* invasion. For instance, expression of members of 13 miRNA families (*e.g.*, miR529, miR390, miR166, and miR160) was significantly induced in the susceptible line, but their levels were reduced or not much changed in the resistant line upon pathogen infection. Hence, they may negatively regulate maize immunity against FER. This observation suggests that some miRNAs suppress plant defense responses in the absence of a pathogen challenge, while a healthy plant can deploy immune responses by attenuating their suppression upon infection [33], [34]. By contrast, levels of just a handful of miRNAs (*e.g.*, miR171d/e/i/j, miR167b, miR164h, and several predicted miRNAs) were down-regulated or not obviously changed in the susceptible line but were significantly up-regulated in the resistant line upon *F. verticillioides* infection. Hence, they may positively regulate maize immunity against FER. Expression of members of some miRNA families was induced in both maize genotypes, suggesting that they may play fundamental roles in maize immunity against *F. verticillioides* infection. Despite sharing highly similar sequences, members of the same miRNA family responded differently to pathogen infection (Table S1, Sheet 2), implying that they might positively or negatively regulate the FER-responsive gene expression concurrently. Hence, it is necessary to develop extensive experimental validation approaches to understand the regulatory roles of distinct miRNA–target pairs during attack by a pathogen. Overall, the identified known or novel miRNAs involved in maize FER immunity serve as a good starting point for further exploration of diverse aspects of the molecular mechanisms behind maize resistance to *F. verticillioides*.

In plants, miRNAs function as gene repressors, primarily through cleavage of the target mRNAs, which leads to a negative correlation of expression between miRNAs and their degraded targets. Numerous studies have shown that expression of host miRNAs and their respective targets is either up or down-regulated, and is involved in modulating the plant disease response upon fungal infection [35]. Opposite expression profiles were observed for *F. verticillioides*-responsive miRNAs and their corresponding target genes in both genotypes in the current study (Figure 6, Figure S8), suggesting that the specific pathogen-induced alterations in the levels of these miRNAs shape the plant transcriptome. However, this inverse relationship was not observed for many miRNA–mRNA pairs proposed in the current study by psRNATarget prediction or degradome analysis, possibly because the transcription profiles of target genes were determined at only one time point in the *F. verticillioides* treatment. In addition, miRNAs also influence gene expression via a translational repression mechanism, which has a minor effect on the expression levels of target mRNAs, as manifested by the observation that a series of potential miRNA targets undergo translational repression events (Table S3).

Functional analysis revealed that in the current study, the predicted miRNA targets were found to encompass diverse biological processes related to plant immunity, particularly the phenylpropanoid metabolic process (Figure S8), implying a close relationship between phenylpropanoid levels in the kernel pericarp and FER symptoms, as has been suggested previously [36]. Hence, it may be worthwhile to investigate the accumulation of phenylpropanoid compounds in susceptible and resistant maize. Our data further suggest a potential role for the miR168–AGO1 pair acting in the miRNA functional pathway, thus coordinating the response of maize to fungal pathogen infection. A number of the miRNA–target pairs predicted by psRNATarget in the current study are likely to be false positives. However, some recently evolved miRNAs might exist without specific target genes [37]. Determination of these predicted miRNA–target partners will be indispensable for future work. In addition, a set of potential novel miRNAs among the *F. verticillioides*-responsive miRNAs identified in the current study may provide a rich source of expression data for future investigations. As key modulators of post-transcriptional regulation, miRNAs provide quantitative regulation rather than an on–off regulation. Hence, the dynamic expression of miRNAs upon fungal infection could allow for the fine-tuning of gene expression of different physiological processes. Therefore, this may enhance the plant’s capacity to overcome diseases.

Molecular mechanisms underlying FER resistance had been explored previously by comprehensive transcriptome analysis in maize, suggesting that both PTI- and ETI-triggered defense responses are involved in immunity against *F. verticillioides* infection [7], [8], [9]. A number of pathogen-responsive miRNAs identified in the current study, including 68 known members of 17 families and 21 predicted miRNAs, were found to be involved in the plant–pathogen interaction process (Figure 5). The activation of MAPK signaling cascades triggered by FLS2 and EFR is responsible for the induction of defense-related genes for the biosynthesis of antimicrobial compounds. Accordingly, expression levels of the respective signaling components targeted by several miRNAs displayed obvious differences between the resistant and susceptible genotypes upon *F. verticillioides* infection. For instance, levels of miR159j and miR166b, targeting the *MEKK1* and *WRKY22*, respectively, were significantly increased in the susceptible genotype but decreased in the resistant genotype upon *F. verticillioides* infection, implying that the MAPK signaling pathway was specifically activated in the resistant genotype but blocked in the susceptible line. In parallel, a specific expression of calcium-dependent protein kinase (*CDPK*) genes controlling ROS production is induced through Ca^2+^ signaling after infection, and thus reinforcing the cell wall structure [38], [39]. Consistently, several miRNA families with significant expression in the susceptible and resistant genotypes (*e.g.*, miR159, miR169, miR160, and miR393) were found to regulate key genes in this signaling cascade. Furthermore, many significant miRNAs in the two genotypes (*e.g.*, miR166, miR408, and miR171) were also involved in the ETI signaling pathway (Figure 5). These observations revealed that the differentially expressed miRNAs identified in the current study serve as positive or negative regulators in maize immunity against *F. verticillioides* infection.

Previous studies have revealed that plant defense responses are regulated through highly complex signaling networks [28], [40]. Some miRNAs are crucial regulators in the innate immunity of plants by modulating downstream hormone signaling pathways [34], [41], [42]. Hence, it is not surprising that a series of pathogen-responsive miRNAs identified in the current study are involved in plant hormone signaling pathways (Figure S7). For instance, the levels of miR396a/b were not changed in the susceptible genotype but were significantly decreased in the resistant genotype after infection, likely resulting in disease resistance along with increased SA levels. Similarly, expression of the miR319 family members was significantly repressed in the resistant line but did not change obviously in the susceptible line upon *F. verticillioides* infection (Table S1, Sheet 2), implying that the jasmonic acid (JA) signaling pathway was anomalously activated, thus leading to resistance. Plants have evolved a mechanism of suppressing auxin signal transduction as a critical part of the basal defense to prevent the invading pathogen from using this hormone as a virulence factor [15], [43]. Correspondingly, expression of the miR164 and miR171 family members, which are basal regulators of plant defenses that regulate auxin and cytokinin signaling cascades, was induced in both genotypes upon infection (Table S1, Sheet 2). In addition to regulating resistance responses, phytohormones synergistically or antagonistically control plant growth, development, and responses to environmental factors [44]. Considering the critical role of hormones in balancing growth and resistance responses [45], it is therefore reasonable to observe that the components of the same hormone-signaling pathway are positively or negatively regulated by miRNAs identified in the current study.

Transgenic experiments have revealed that host miRNAs play critical roles in plant immunity, *e.g.*, miR393, miR160, miR166, miR398, and miR528 [15], [16], [19], [25]. In the current study, we have shown that the ectopic accumulation of miR408b contributes to a phenotype of increased susceptibility to *F. verticillioides* in the susceptible genotype (Figure 7)*.* This finding supports a negative role for miR408b in FER resistance, most likely associated with control of the target genes. Previous reports indicated that miR408 was also involved in fungal disease resistance in wheat. Differential regulation of miR408 in wheat cultivars results in susceptibility or resistance to *Puccinia graminis* [35]. Further, Feng et al [46] reported that tae-miR408 may play a positive role in wheat resistance against stripe rust as well as abiotic stresses via mediation expression of its target gene encoding chemocyanin-like protein (TaCLP1). Genes encoding plantacyanin and several laccases are well-known targets of miR408 in plants [47]. Although the exact physiological functions of plantacyanins in plants are not entirely clear yet, it is assumed that plantacyanins act in stress responses, lignin production, and cell to cell signaling [47]. Similarly, plant laccases have been reported to regulate diverse functions in plants, such as lignin synthesis, wound healing, plant cell wall integrity maintenance, and stress responses [48]. Therefore, dynamic expression of miR408 in susceptible and resistant maize genotypes upon *F. verticillioides* infection may result in plantacyanins- or laccase-mediated perturbance of lignin biosynthesis, and a resultant hypersensitivity response to reduce epidermis rupture. Consequently, future research efforts should be directed at delineating the control of cell wall structure or lignin accumulation by miR408 target genes in FER-susceptible and resistant maize.

miR408 is one of the most evolutionarily conserved miRNA families across plant species [49], suggesting that it could be a potential candidate for developing fungal disease-resistant plants, including various crops. It has been shown that miR408 positively regulates growth, biomass, and seed yield in different plants [50], [51], and accumulation of miR408 results in sensitivity to drought and osmotic stress [52]. This suggests that the regulation of miR408 under different biotic and abiotic stresses might cause alteration of expression of a specific target gene. Hence, it would be interesting to investigate how maize resistance to *F. verticillioides* is reconciled by individual miR408–target pairs.

To conclude, a set of miRNAs responsive to *F. verticillioides* infection was identified in two inbred maize lines, which acts either positively or negatively to regulate maize immunity against the fungus via integrated miRNA-mediated regulatory networks. Ectopic expression of a single miRNA altered maize susceptibility to FER. miRNA functional analysis implied that genetic or induced variation of miRNA expression could be applied to breeding programs aimed at enhancing FER resistance in maize.

## Materials and methods

### Plant material and *F. verticillioides* inoculation

Two inbred maize lines of N6 and BT-1 with contrasting FER resistance were used in the current study, which show susceptibility and resistance to FER, respectively, as described previously [9]. For the RNA sequencing experiments, kernels of the two inbred lines 15 d after pollination were inoculated with a single *F. verticillioides* strain (1 × 10^5^ spores/ml suspension) under field growth conditions in the years 2015 and 2016 and grown at the Zhengzhou Experiment Station (34°510′N 113°350′E). The kernels closely surrounding the inoculation spots were harvested from five individual maize plants and pooled, as shown in Figure 1, on days 1 or 3 after inoculation and placed immediately in liquid nitrogen.

### Small RNA library construction and sequencing

Total RNA was extracted from three mixed independent samples, each comprising six kernels close to the inoculation spot using a plant RNA Easyspin isolation kit (Catalog no. RN38, Aidlab Biotech, Beijing, China) following the manufacturer’s instructions. RNA purity, concentration, and integrity were determined by a Nanodrop, Qubit 2.0, and an Agilent 2100 bioanalyzer, respectively. Small RNA libraries were then constructed and subjected to high-throughput sequencing on the Illumina Hiseq2500 platform (BIOMARKER, Beijing, China). Paired-end reads were generated.

### Identification of maize miRNAs

Raw sequencing reads were firstly processed through in-house Perl scripts to obtain clean reads by removing reads containing adapter, containing ploy-N and low quality reads. The length distribution of clean reads was then categorized to analyze the composition of small RNA data, and 18- to 30-nt-long small RNAs were used for further analysis. High-quality clean reads were mapped to the maize reference genome (ZmB73_RefGen_v4) by SOAP (short oligonucleotide alignment program) to detect their expression and distribution in the genome. The matched clean reads were then aligned with information in the Silva, GtRNAdb, Rfam, and Repbase databases to filter rRNA, tRNA, snRNA, snoRNA, other ncRNAs, and repeats, respectively. The remaining reads were used to detect known miRNAs, and new miRNAs were predicted by comparing with known miRNAs from the miRBase release v21 (on June 26, 2014), allowing no mismatches. miRDeep2 was used to identify novel miRNAs based on criteria outlined [53]. Randfold software was used to check for their corresponding precursor sequences to ensure that the miRNA precursors have the expected secondary structures. The IDEG6 package was used to detect differential miRNA expression between two samples with a criterion of fold change ≥ 1.5 and adjusted *P* < 0.05 [54]. miRNA expression was quantified as reads per million (RPM).

### Small RNA gel-blot analysis

RNA gel blots were performed to determine the expression levels of known miRNAs and characterize predicted new miRNAs. Approximately 30 μg total RNA was separated by electrophoresis in a 12% urea polyacrylamide gel, transferred to a Hybond-N^+^ membrane and UV cross-linked. Probes were labeled and blots were hybridized as previously described [50]. Probe sequences are listed in Table S5.

### RT-qPCR

Reverse transcription was carried out with DNase I-treated total RNA using a Super-Script II reverse transcription kit (Catalog No. 11755050, Invitrogen, Shanghai, China) for detection of protein-coding genes. For quantification of mature miRNA molecules, the total RNA sample was first polyadenylated and then reversely transcribed by using the Ncode miRNA first-strand cDNA synthesis kit (Catalog No. MIRC-10, Invitrogen, Carlsbad, CA). Stem-loop reverse-transcription was performed using a TaqMan_MicroRNA Reverse Transcription kit (Catalog No. 4366596, Applied Biosystems, Pleasanton, CA) to validate the predicted miRNA precursors. The resulting cDNA was used for qPCR with the Power SYBR Green PCR master mix (Takara, Dalian, China). Gene expression levels were normalized using 5S rRNA as an internal control. Data were generated with three biological replications with three technical replications of each. The relative expression levels of mRNAs or miRNAs were determined by using the 2^−ΔΔC(T)^ comparative method. The primer sequences are listed in Table S5.

### Degradome and mRNA transcriptome analysis

For degradome sequencing, total RNA was first extracted from each sample, and mRNAs were isolated using oligo-dT bead extraction according to the manufacturer’s instructions (Catalog No. Z5210, Promega, Madison, WI). The purified mRNAs were pooled together. Degradome sequencing was performed using an Illumina Hiseq2500 platform at BIOMARKER (Beijing, China). We used the CleaveLand 3.0 pipeline [55] to identify and classify putative sliced miRNA targets. The sequences were regarded as valid targeting events based on the following criteria: (1) CleaveLand category in 0, 1, and 2, (2) ≥ 5 reads at the predicted cleavage site, (3) the adjusted *P* < 0.05.

Transcriptome in RNA samples from the control and 3 DPI kernels of the susceptible and resistant genotypes, the same as those used for small RNA sequencing, was then sequenced on the Illumina Hiseq2500 platform at BIOMARKER. Data processing and identification of gene expression were performed as previously described [9], using the reference genome ZmB73_RefGen_v4.

### Promoter analysis of *F. verticillioides*-responsive miRNAs

For each set of differentially expressed miRNAs, the promoter sequences (2 kb upstream) of all miRNAs were extracted from the maize B73 reference genome (ZmB73_RefGen_v4). The motifs in the promoter sequences were identified using the Arabidopsis motif database (DAP and PBM motifs) and MAST (version 4.12.0), a component of the MEME suite [56]. To assign roles to the identified motifs, the motifs were provided as input files of GOMO (version 4.12.0), which had been developed to identify GO terms significantly associated with specified motifs [57]. For each association, the score, *P* value, and specificity were provided.

### Construction of miR408b-overexpressing transgenic maize

To construct the zma-miR408b overexpression plasmid, the genomic sequence from 200 bp upstream to160 bp downstream of the miR408b precursor was amplified using N6 gDNA. PCR products were inserted into the binary vector pFGC5941 (Biovector, Beijing, China) at the *Nco*I/*Xba*I site. The resultant construct was transformed to the susceptible inbred N6 line by *Agrobacterium tumefaciens*-mediated transgenic introduction. Transgenic maize plants were screened by PCR amplification of the Basta resistant gene (*Bar*) and a specific inserted DNA fragment, using the forward primer 35F and the specific reverse primer zma-miR408b-RP. T2 transgenic plants were used for further analysis. Primers used for plasmid construction and PCR identification are listed in Table S5.

### Statistical analysis

Two-tailed Student’s *t*-tests were performed to determine statistical significance by comparing the mean values from three independent samples. The differences were deemed significant at *P* < 0.05.

## Data availability

The raw sequence data of small RNA, transcriptome, and degradome reported in the current paper have been deposited in the Genome Sequence Archive [58] at the Beijing Institute of Genomics, Chinese Academy of Sciences / China National Center for Bioinformation (GSA: CRA001239), and are publicly accessible at http://bigd.big.ac.cn/gsa.

## CRediT author statement

**Zijian Zhou:** Investigation, Resources, Validation, Formal analysis. **Yan Cao:** Investigation, Resources, Validation, Formal analysis. **Tao Li:** Investigation, Validation, Visualization. **Xinghao Wang:** Investigation, Validation. **Jiafa Chen:** Resources. **Hang He:** Software. **Wen Yao:** Software, Visualization. **Jianyu Wu:** Conceptualization, Resources, Funding acquisition. **Huiyong Zhang:** Conceptualization, Methodology, Project administration, Supervision, Writing-original draft, Writing-review and editing, Visualization, Funding acquisition. All authors read and approved the final manuscript.

## Competing interests

The authors have declared no competing interests.

## Supplementary material

Supplementary Figure S1 **Profiling of unique small RNAs from different sample libraries of the susceptible and resistant maize kernels A.–C**. Length distribution of sequenced small RNAs uniquely mapped to the maize genome (A), the known miRNA precursors (B), and the predicted novel miRNAs (C). **D.** Number of detected miRNA family members in the six small RNA libraries.

Supplementary Figure S2 **Validation of the expressed miRNAs from small RNA sequencing datasets A.** Accumulation of miR166, miR156, miR398b, and miR529 in maize kernels. Northern blot analysis was performed to evaluate the correlation between the hybridization signal and relative abundance of distinct miRNAs (as determined by sequencing; numbers in parenthesis indicate the relative abundance of each miRNA in the control samples). For the experiment, 30 μg of total RNA was loaded per lane. The exposure time was the same for all four hybridization experiments. **B.–D.** Detection of the predicted miRNAs. Experimental confirmation of the predicted miRNAs by stem-loop RT-PCR (B) and RNA blotting (C), and the predicted hairpin structure of the confirmed novel miRNAs, as indicated by the RNAfold web server, with the positions of mature miRNA sequences indicated (D).

Supplementary Figure S3 **Expression profiles of *F. verticillioides*-responsive miRNAs were grouped according to model profiles (black line) by STEM analysis** For each of the 50 profiles per sample, the expression pattern of individual miRNA (red lines) across 6 tested samples (plotted equally along the *x*-axis) is plotted using log2 normalized data (each *y*-axis tick represents two log2 intervals of normalized miRNA reads). Each model profile is anchored at 0 for N6 (CK) sample. Colored plots represent model profiles for which there was an excess (*P* < 0.005) of assigned versus expected miRNA reads. **A**, N6 (CK); **B**, N6 (1 DPI); **C**, N6 (3 DPI); **D**, BT-1 (CK); **E**, BT-1 (1 DPI); **F**, BT-1 (3 DPI).

Supplementary Figure S4 **Comparison of differentially expressed miRNAs (DEMs) between the susceptible and resistant genotypes in the absence or presence of pathogen inoculation A.** Statistical analysis of miRNAs that were differentially expressed between N6 and BT-1 genotypes in the CK, 1 DPI, and 3 DPI samples. **B.** Distribution of the significantly expressed miRNAs in the control, 1 DPI, and 3 DPI samples. **C.** RT-qPCR validation of randomly selected *F. verticillioides*-responsive miRNAs in the susceptible and resistant genotypes. CK, control; DPI, day post inoculation.

Supplementary Figure S5 **Distribution of differentially expressed miRNAs in the FER-resistance metaQTL in maize A.***F. verticillioides*-responsive miRNAs identified in the susceptible and resistant maize lines. **B.** Differentially expressed miRNAs between the susceptible and resistant maize lines in the CK, 1 DPI, and 3 DPI samples. The short horizontal lines in various colors represent the locations of anti-ear rot QTLs determined previously, with the associated PMID shown. The short vertical lines represent the different DEMs. FER, *F. verticillioides* ear rot; CK, control; DPI, day post inoculation.

Supplementary Figure S6 **Expression of miRNAs and their target genes involved in the plant–pathogen interaction in the susceptible or resistant maize inbred lines A.** RT-qPCR analysis showing that *F. verticillioides*-responsive miRNAs (upper panel) identified in the current study and their potential targets (lower panel) are differently regulated in the susceptible maize genotype. **B.** RT-qPCR analyss of miRNAs and their potential target genes in the resistant maize genotypes, as presented in panel A. Maize inbred lines N6, TSPT21, and TSPT12 are susceptible to *F. verticillioides* infection, and inbred lines BT-1, CML533, and CML325 are resistant to *F. verticillioides* infection. Kernels of various genotypes 15 days after pollination were inoculated with an *F. verticillioides* spore suspension (1 × 105 spores/ml), and kernels close to the inoculation spots were collected at 3 DPI. RNA was extracted for RT-qPCR analysis, and miRNA or mRNA levels were normalized to those in an untreated control sample. WRKY22, Zm00001d008578; PTI1, Zm00001d010919; RPM1, Zm00001d014654. The experiments were repeated three times, with similar results. *, *P* < 0.05; **, *P* < 0.001 (two-tailed Student’s *t*-test).

Supplementary Figure S7 **Maize miRNAs are involved in the plant hormone signaling pathways** The plant hormone signal transduction was downloaded from the website (https://www.genome.jp/kegg-bin/show_pathway?zma04075), in which genes encoding the key components (green-colored) were explored to be potentially targeted by the corresponding miRNAs identified in the present study. Known miRNAs are denoted in red and predicted new miRNAs are indicated in blue.

Supplementary Figure S8 **Functional annotation of FER resistance-associated miRNAs targets by psRNATarget A.** Enriched representative GO terms in the biological process category that are associated with the target genes. **B.** Enriched representative pathways associated with the target genes by KEGG (Kyoto encyclopedia of genes and genomes) analysis. The corresponding *P* values are indicated.

Supplementary Figure S9 **Correlated expression of *F. verticillioides*-responsive miRNAs and their potential target genes** Kernels of the susceptible (**A**) or resistant (**B**) lines with (3 DPI) or without *F. verticillioides* inoculation were analyzed by RNA sequencing, and the target genes were predicted by combining the degradome and psRNATarget data. DPI, day post inoculation.
