# Supplementary figures and images for: MicroRNAs Are Involved in Maize Immunity Against Fusarium verticillioides Ear Rot
Source: Genomics Proteomics Bioinformatics. 2020 Jun 10;18(3):241–55. doi: 10.1016/j.gpb.2019.11.006 (PMC7801212; doi:10.1016/j.gpb.2019.11.006)

**A Unique reads**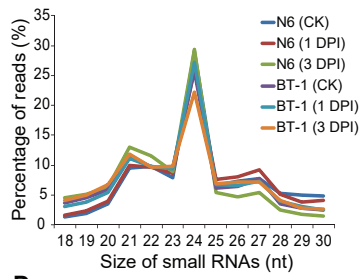**B Known miRNAs**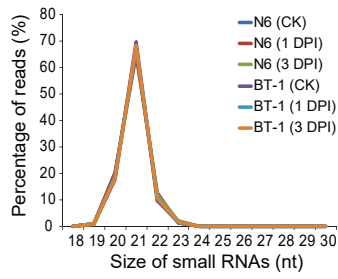**C Predicted miRNAs**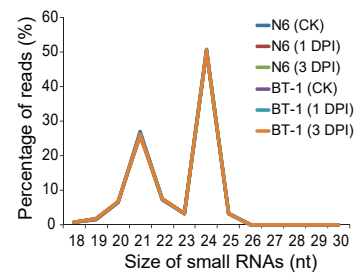**D**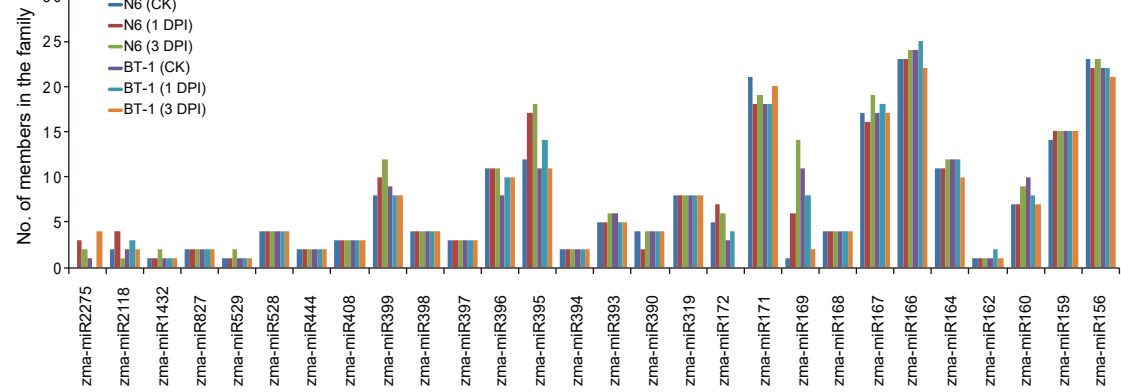

Supplement: Supplementary Figure S1 — Profiling of unique small RNAs from different sample libraries of the susceptible and resistant maize kernels A.–C. Length distribution of sequenced small RNAs uniquely mapped to the maize genome (A), the known miRNA precursors (B), and the predicted novel miRNAs (C). D. Number of detected miRNA family members in the six small RNA libraries. [file mmc1.pdf]

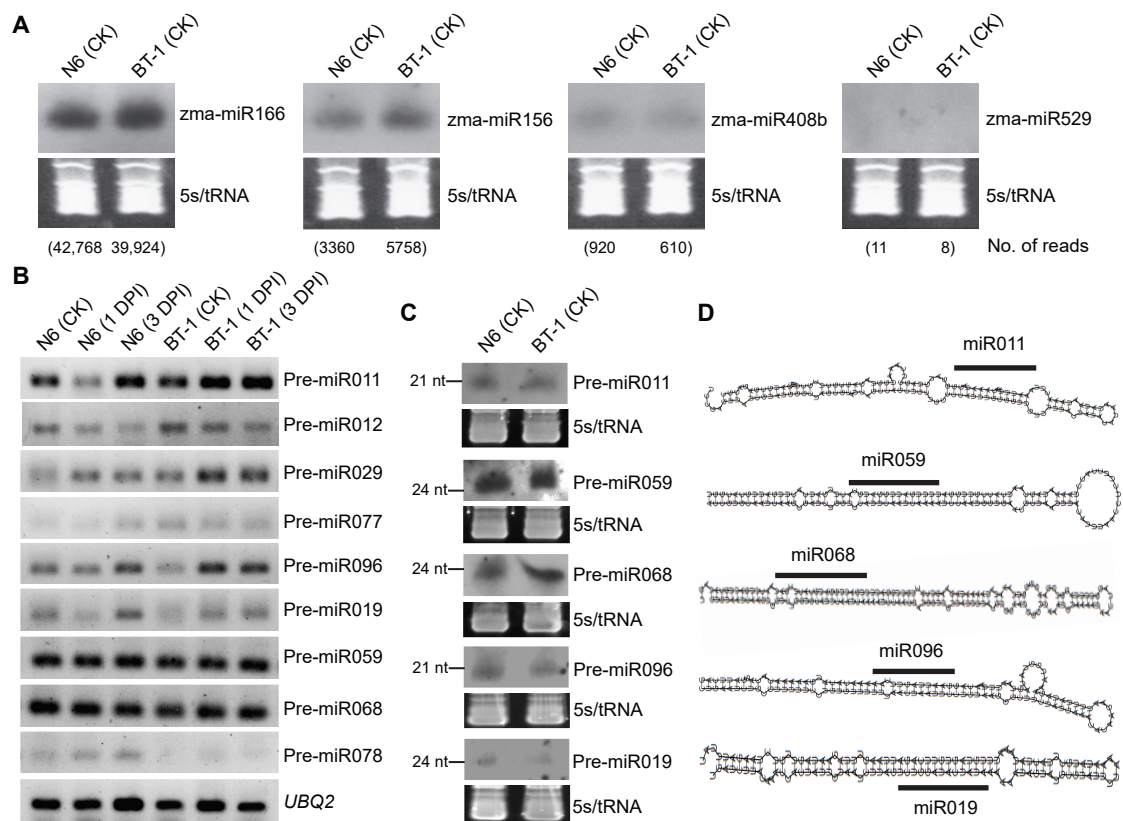

Supplement: Supplementary Figure S2 — Validation of the expressed miRNAs from small RNA sequencing datasets A. Accumulation of miR166, miR156, miR398b, and miR529 in maize kernels. Northern blot analysis was performed to evaluate the correlation between the hybridization signal and relative abundance of distinct miRNAs (as determined by sequencing; numbers in parenthesis indicate the relative abundance of each miRNA in the control samples). For the experiment, 30 μg of total RNA was loaded per lane. The exposure time was the same for all four hybridization experiments. B.–D. Detection of the predicted miRNAs. Experimental confirmation of the predicted miRNAs by stem-loop RT-PCR (B) and RNA blotting (C), and the predicted hairpin structure of the confirmed novel miRNAs, as indicated by the RNAfold web server, with the positions of mature miRNA sequences indicated (D). [file mmc2.pdf]

Log<sub>2</sub> normalized expression levels

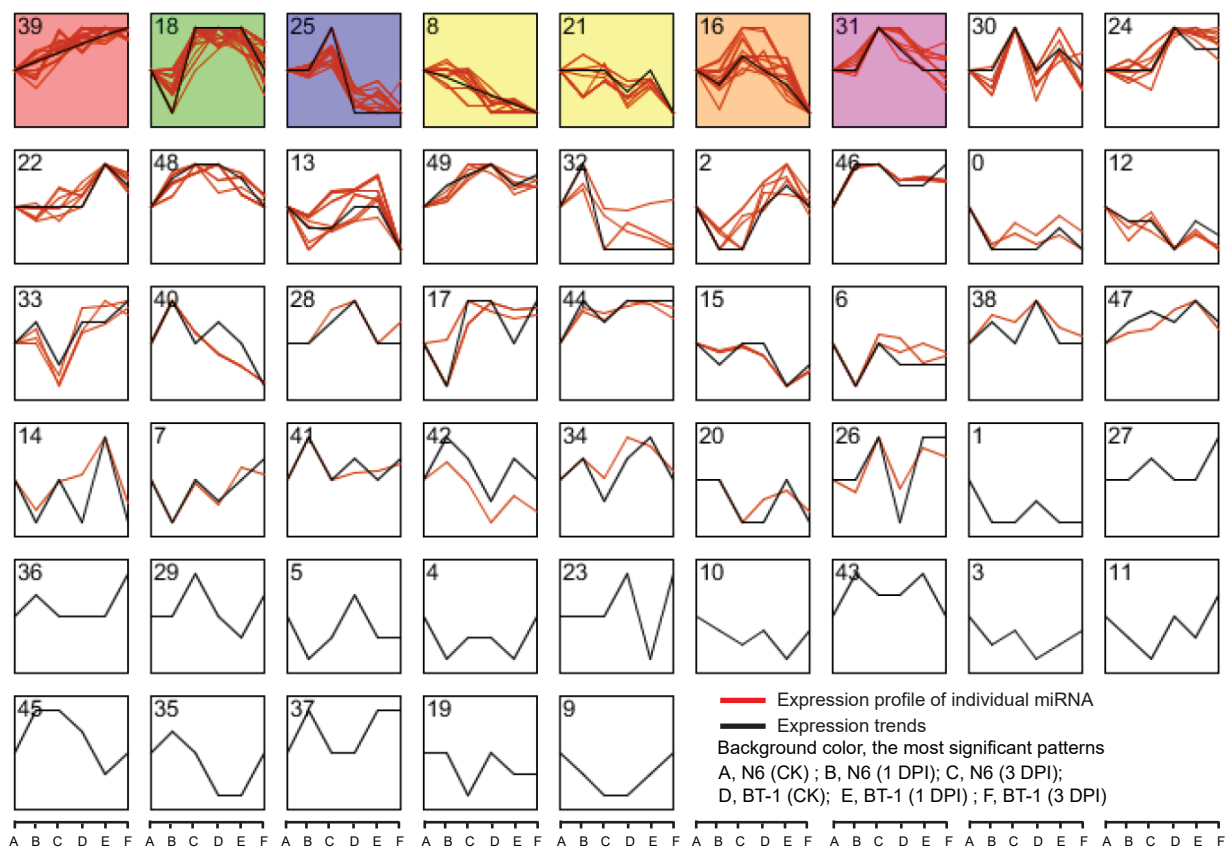

Supplement: Supplementary Figure S3 — Expression profiles of F. verticillioides-responsive miRNAs were grouped according to model profiles (black line) by STEM analysis For each of the 50 profiles per sample, the expression pattern of individual miRNA (red lines) across 6 tested samples (plotted equally along the x-axis) is plotted using log2 normalized data (each y-axis tick represents two log2 intervals of normalized miRNA reads). Each model profile is anchored at 0 for N6 (CK) sample. Colored plots represent model profiles for which there was an excess (P < 0.005) of assigned versus expected miRNA reads. A, N6 (CK); B, N6 (1 DPI); C, N6 (3 DPI); D, BT-1 (CK); E, BT-1 (1 DPI); F, BT-1 (3 DPI). [file mmc3.pdf]

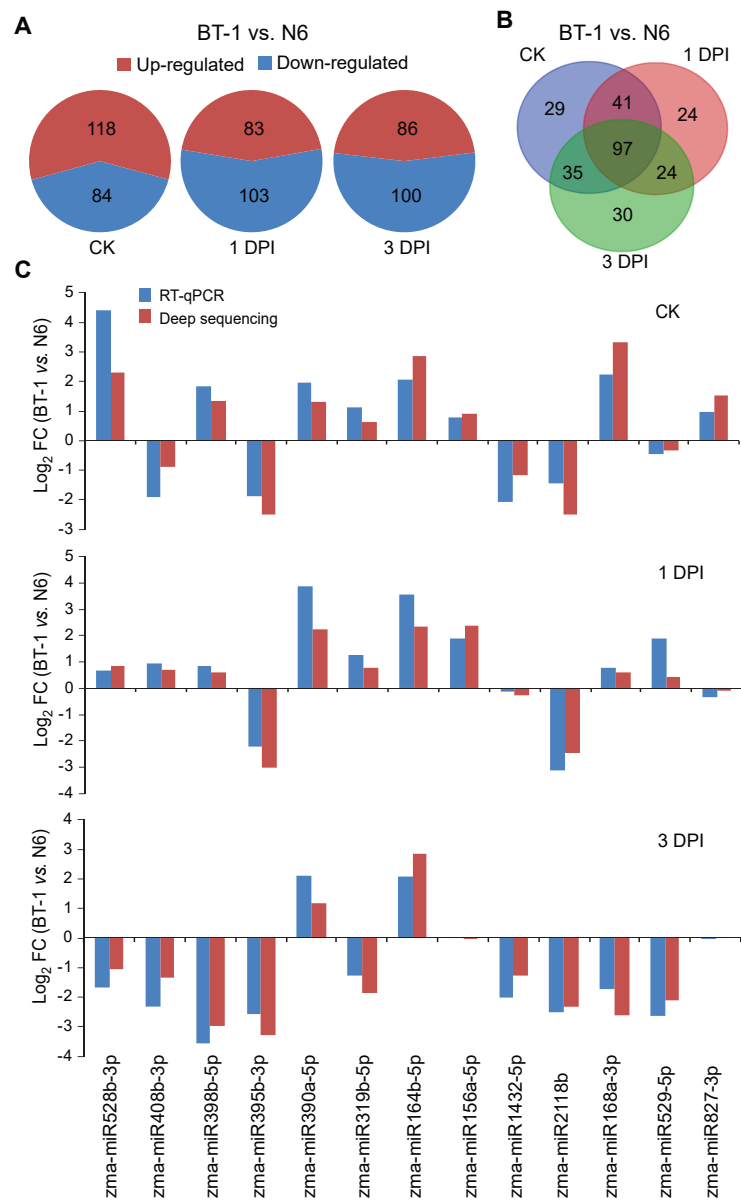

Supplement: Supplementary Figure S4 — Comparison of differentially expressed miRNAs (DEMs) between the susceptible and resistant genotypes in the absence or presence of pathogen inoculation A. Statistical analysis of miRNAs that were differentially expressed between N6 and BT-1 genotypes in the CK, 1 DPI, and 3 DPI samples. B. Distribution of the significantly expressed miRNAs in the control, 1 DPI, and 3 DPI samples. C. RT-qPCR validation of randomly selected F. verticillioides-responsive miRNAs in the susceptible and resistant genotypes. CK, control; DPI, day post inoculation. [file mmc4.pdf]

## A FER-susceptible inbred lines

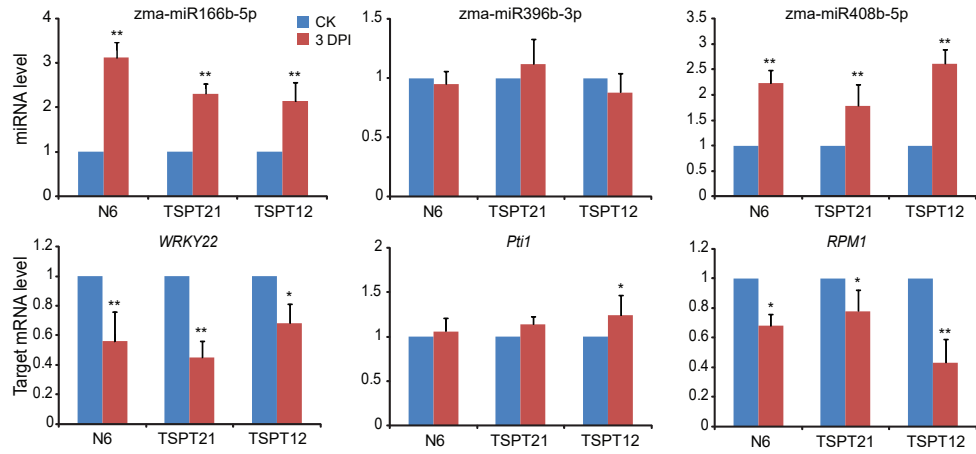

## B FER-resistant inbred lines

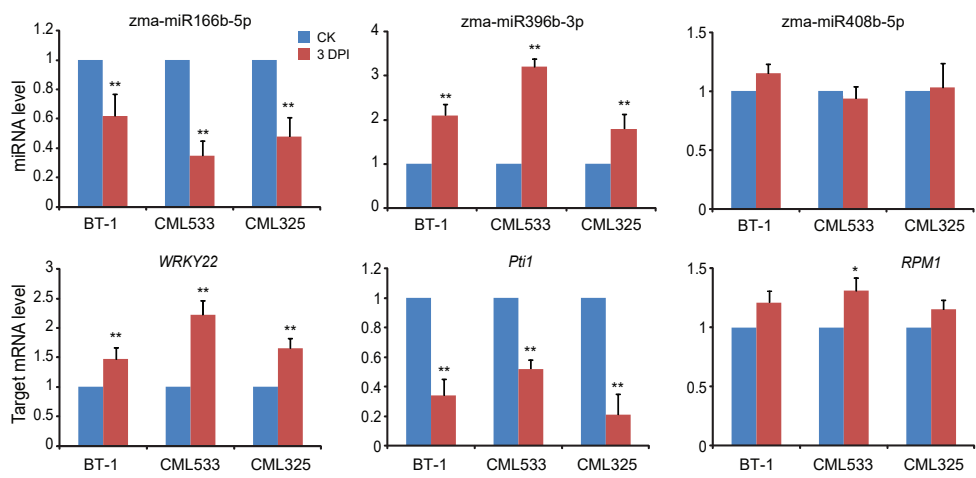

Supplement: Supplementary Figure S6 — Expression of miRNAs and their target genes involved in the plant–pathogen interaction in the susceptible or resistant maize inbred lines A. RT-qPCR analysis showing that F. verticillioides-responsive miRNAs (upper panel) identified in the current study and their potential targets (lower panel) are differently regulated in the susceptible maize genotype. B. RT-qPCR analyss of miRNAs and their potential target genes in the resistant maize genotypes, as presented in panel A. Maize inbred lines N6, TSPT21, and TSPT12 are susceptible to F. verticillioides infection, and inbred lines BT-1, CML533, and CML325 are resistant to F. verticillioides infection. Kernels of various genotypes 15 days after pollination were inoculated with an F. verticillioides spore suspension (1 × 10^5^ spores/ml), and kernels close to the inoculation spots were collected at 3 DPI. RNA was extracted for RT-qPCR analysis, and miRNA or mRNA levels were normalized to those in an untreated control sample. WRKY22, Zm00001d008578; PTI1, Zm00001d010919; RPM1, Zm00001d014654. The experiments were repeated three times, with similar results. *, P < 0.05; **, P < 0.001 (two-tailed Student’s t-test). [file mmc6.pdf]

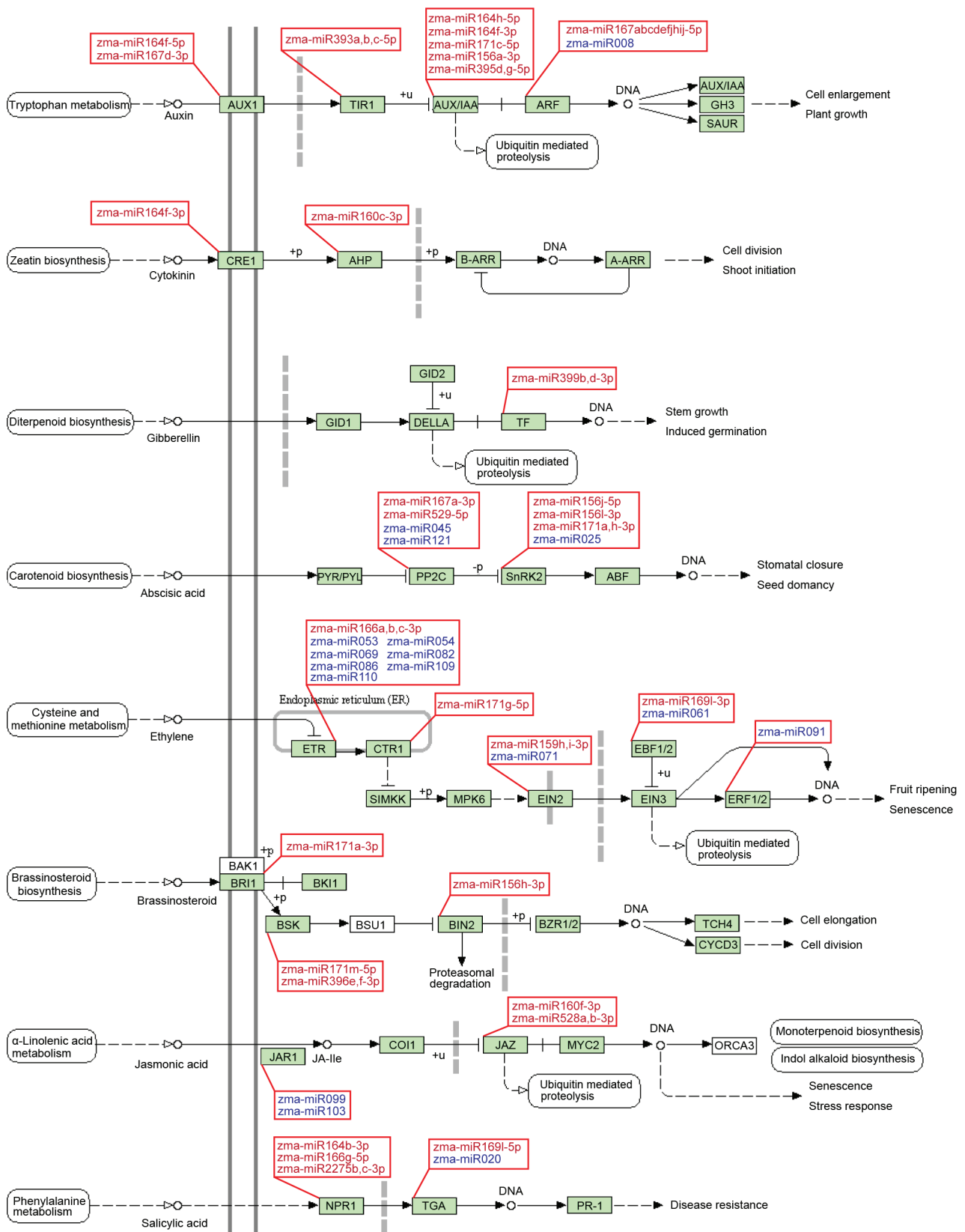

Supplement: Supplementary Figure S7 — Maize miRNAs are involved in the plant hormone signaling pathways The plant hormone signal transduction was downloaded from the website (https://www.genome.jp/kegg-bin/show_pathway?zma04075), in which genes encoding the key components (green-colored) were explored to be potentially targeted by the corresponding miRNAs identified in the present study. Known miRNAs are denoted in red and predicted new miRNAs are indicated in blue. [file mmc7.pdf]

### A GO annotation

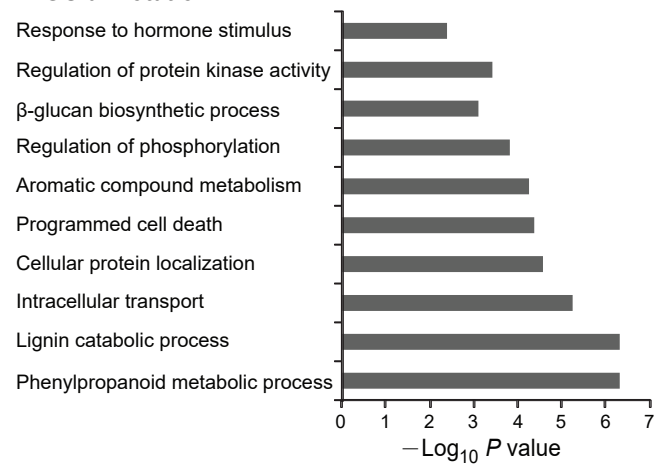

### B KEGG annotation

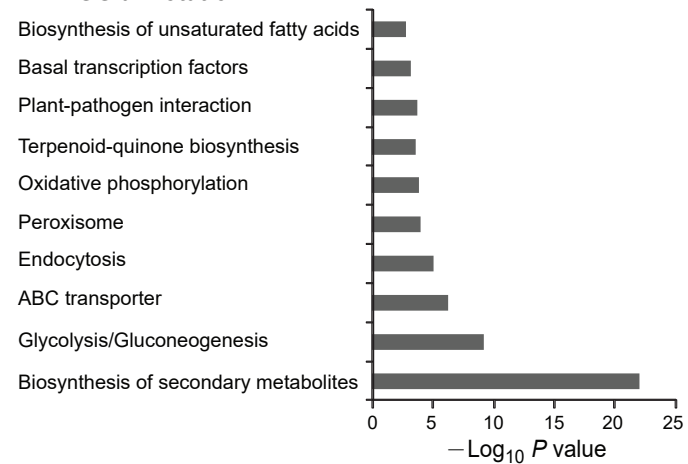

Supplement: Supplementary Figure S8 — Functional annotation of FER resistance-associated miRNAs targets by psRNATarget A. Enriched representative GO terms in the biological process category that are associated with the target genes. B. Enriched representative pathways associated with the target genes by KEGG (Kyoto encyclopedia of genes and genomes) analysis. The corresponding P values are indicated. [file mmc8.pdf]

**A N6 susceptible line**

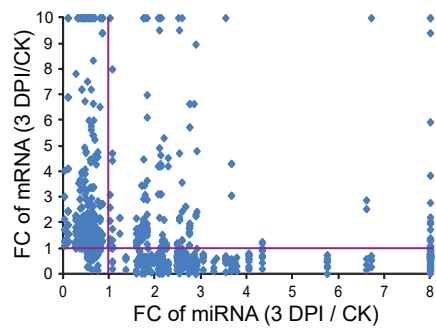

**B BT-1 resistant line**

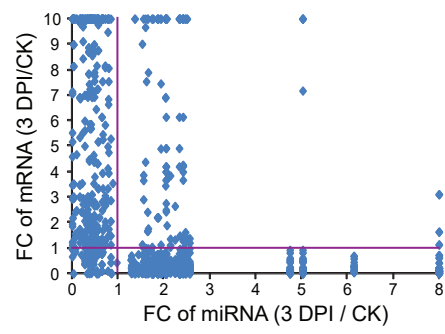

Supplement: Supplementary Figure S9 — Correlated expression of F. verticillioides-responsive miRNAs and their potential target genes Kernels of the susceptible (A) or resistant (B) lines with (3 DPI) or without F. verticillioides inoculation were analyzed by RNA sequencing, and the target genes were predicted by combining the degradome and psRNATarget data. DPI, day post inoculation. [file mmc9.pdf]
