# Supplementary material for: MicroRNAs Are Involved in Maize Immunity Against Fusarium verticillioides Ear Rot
Source: Genomics Proteomics Bioinformatics. 2020 Jun 10;18(3):241–55. doi: 10.1016/j.gpb.2019.11.006 (PMC7801212; doi:10.1016/j.gpb.2019.11.006)
Supplement: Supplementary Figure S5 — Distribution of differentially expressed miRNAs in the FER-resistance metaQTL in maize A.F. verticillioides-responsive miRNAs identified in the susceptible and resistant maize lines. B. Differentially expressed miRNAs between the susceptible and resistant maize lines in the CK, 1 DPI, and 3 DPI samples. The short horizontal lines in various colors represent the locations of anti-ear rot QTLs determined previously, with the associated PMID shown. The short vertical lines represent the different DEMs. FER, F. verticillioides ear rot; CK, control; DPI, day post inoculation. [file mmc5.pdf]

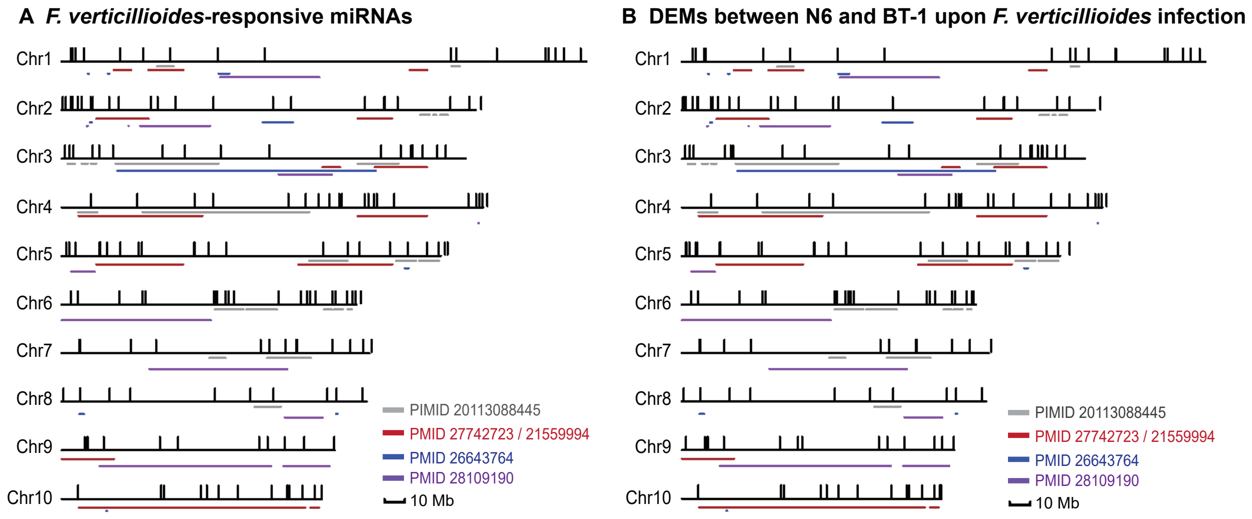
A F. verticillioides-responsive miRNAs
B DEMs between N6 and BT-1 upon F. verticillioides infection
Chr1
Chr2
Chr3
Chr4
Chr5
Chr6
Chr7
Chr8
Chr9
Chr10
PIMID 20113088445
PMID 27742723 / 21559994
PMID 26643764
PMID 28109190
10 Mb
